# Author Correction: Hydrogen-based metabolism as an ancestral trait in lineages sibling to the Cyanobacteria

**DOI:** 10.1038/s41467-019-09423-3

**Published:** 2019-03-26

**Authors:** Paula B. Matheus Carnevali, Frederik Schulz, Cindy J. Castelle, Rose S. Kantor, Patrick M. Shih, Itai Sharon, Joanne M. Santini, Matthew R. Olm, Yuki Amano, Brian C. Thomas, Karthik Anantharaman, David Burstein, Eric D. Becraft, Ramunas Stepanauskas, Tanja Woyke, Jillian F. Banfield

**Affiliations:** 1 Department of Earth and Planetary Science, University of California, Berkeley, Berkeley, 94720 CA USA; 2 DOE Joint Genome Institute, Walnut Creek, 94598 CA USA; 3 Feedstocks Division, Joint BioEnergy Institute, Emeryville, 94608 CA USA; 4 Environmental Genomics and Systems Biology Division, Lawrence Berkeley National Laboratory, Berkeley, 94720 CA USA; 5 Institute of Structural & Molecular Biology, Division of Biosciences, University College London, London, WC1E 6BT UK; 6 Department of Plant and Microbial Biology, University of California, Berkeley, Berkeley, 94720 CA USA; 7 Nuclear Fuel Cycle Engineering Laboratories, Japan Atomic Energy Agency, Tokai, 319-1111 Ibaraki Japan; 8 Horonobe Underground Research Center, Japan Atomic Energy Agency, Horonobe, 098-3224 Hokkaido Japan; 9 Bigelow Laboratory for Ocean Sciences, East Boothbay, 04544 ME USA; 10 Chan Zuckerberg Biohub, San Francisco, 94158 CA USA; 11 Earth Sciences Division, Lawrence Berkeley National Laboratory, Berkeley, 94705 CA USA; 12 Innovative Genomics Institute, Berkley, 94704 CA USA; 13 Present Address: Department of Civil and Environmental Engineering, University of California, Berkeley, Berkeley, 94720 CA USA; 14 Present Address: Department of Plant Biology, University of California, Davis, Davis, 95616 CA USA; 15 Migal Galilee Research Institute, Kiryat Shmona, 11016 Israel; 16 Tel Hai College, Upper Galilee, 12210 Israel; 17 Present Address: Department of Bacteriology, University of Wisconsin-Madison, Madison, 53706 WI USA; 18 Present Address: School of Molecular and Cell Biology and Biotechnology, George S. Wise Faculty of Life Sciences, Tel Aviv University, Tel Aviv, 69978 Israel; 19 Present Address: Department of Biological Sciences, North Alabama University, Florence, 35632 AL USA

Correction to: *Nature Communications* 10.1038/s41467-018-08246-y, published online 28 January 2019

The original version of this Article contained errors in Fig. 4. In panel a, the labels ‘F420-reducing NiFe hydrogenase (group 3a)’ and ‘Group 2 NiFe hydrogenase’ were misplaced. These errors have been corrected in both the PDF and HTML versions of the Article.

