# Supplementary material for: Hydrogen-based metabolism as an ancestral trait in lineages sibling to the Cyanobacteria
Source: Nat Commun. 2019 Jan 28;10:463. doi: 10.1038/s41467-018-08246-y (PMC6349859; doi:10.1038/s41467-018-08246-y)
Supplement: Supplementary file 1 — Supplementary Information [file 41467_2018_8246_MOESM1_ESM.pdf]

Supplementary Information for

Hydrogen-based metabolism as an ancestral trait in lineages sibling to the Cyanobacteria

Matheus Carnevali, P. B., Schulz, F., et al.

This PDF file includes:

Supplementary Methods
Supplementary Notes 1-8
Supplementary Figures 1-5
Supplementary References

Supplementary Methods

Samples collection, DNA extraction, and sequencing

For sampling, DNA processing, sequencing information, metagenome assembly, genome binning and curation of the publicly available aquifer genomes see Brown, et al. [1] and Anantharaman, et al. [2].

Margulisbacteria RA1A was obtained from an acetate amendment experiment (Rifle Acetate Amendment Columns) conducted at Rifle in 2010 [3]. Individual columns packed with sediment (13 and a background sample) were sacrificed from days 13 to 61 to encompass two geochemical processes: iron reduction and sulfate reduction. DNA extraction, Illumina library preparation and DNA sequencing protocols were described elsewhere [3, 4].

Margulisbacteria AA1A and AG2A-AG6A were collected from the Gulf of Maine at a 1 m depth off the coast of Boothbay Harbor, Maine (43.84 N, -69.64 W) on 16 September, 2009 (AAA071) and sorted immediately, targeting small heterotrophic protists [5]. 1 mL samples were collected at a depth from 112 m and 180 m the Southeast Pacific Ocean (-23.46 N, -88.77 E and -26.25 N, -103.96 E for AG-333 and AG-343, respectively) on 1 December, 2010, from the Western Atlantic Ocean at a depth of 100 m (9.55 N, -50.47 E and 24.71 N, -66.80 E for AG-410 and AG-414, respectively) on 1 June, 2010, and at a depth of 72 m in the Eastern Atlantic (17.40 N, -24.50 E) on 1 December, 2011 (AG-439), amended with 10% glycerol and stored at −80°C.

Melainabacteria BJ4A was obtained from the Mizunami Underground Research Laboratory in Japan. For sampling refer to Ino et al. [6] and for DNA processing and sequencing methods refer to Hernsdorf et al. [7]. In brief, groundwater (30 L) was collected in 2014 from a highly fractured bedrock domain (HFDB) in a horizontal borehole (interval #3) part of a 300 m deep stage, by filtration through 0.22 µm GVWP filters. Genomic DNA was extracted using an Extrap Soil DNA Kit Plus version 2 (Nippon Steel and Sumikin EcoTech Corporation), libraries prepared using TruSeq Nano DNA Sample Prep Kit (Illumina), and 150 bp PE reads with a 550 bp insert size were sequenced by Hokkaido System Science Co. using Illumina HiSeq2500 [7].

Four other Melainabacteria genomes (AS1A, AS2A, AS3A, and AS3B) were part of a study of the microbiome in the arsenic-impacted human gut. Faecal samples were obtained from 10 Bangladeshi men (aged between 27-52) from the Laksam Upazila, Bangladesh, that all displayed signs of arsenicosis and were consuming arsenic in their drinking water. Samples were collected on four consecutive days and stored at -20°C until they were shipped to the UK on dry ice. The samples were then stored at -80°C until nucleic acid extraction. DNA was isolated from the faecal samples using PowerFaecal DNA isolation kit (MoBio) according to the manufacturer's instructions and stored at -20°C until they were sent to RTLGENOMICS (Texas, USA) frozen on dry ice. Samples were then prepared using the Kapa HyperPlus Kit (Kapa Biosystems) following the manufacturer's protocol, except that the DNA was fragmented physically using the Diagenode Bioruptor, instead of enzymatically. The resulting individual libraries were run on a Fragment Analyzer (Advanced Analytical) to assess the size distributions of the libraries, quantified using a Qubit 2.0 fluorometer (Life Technologies), and also quantified using the Kapa Library Quantification Kit (Kapa Biosystems). Individual libraries were then pooled equimolar into their respective lanes and loaded onto an Illumina HiSeq 2500 (Illumina, Inc.) 2 x125 flow cell and sequenced.

Data processing, assembly and binning

Margulisbacteria RA1A: The bin for Margulisbacteria RAAC was recovered from the aac11 sample, during the sulfate reduction phase. Reads for each of the 14 RAAC samples were first trimmed using Sickle (https://github.com/najoshi/sickle) and assembled using idba_ud [8] with default parameters. To bin the assembled data we used an abundance-pattern based approach in which scaffolds are clustered

based on their abundance across multiple samples [9] using ESOM [10, 11]. Reads were mapped separately to each of the 14 assemblies using bowtie [12] with parameters -q -n 2 -e 200 -p 6 --best --sam. To prepare the data for ESOM we used version 1.00 of the script prepare_esom_files.pl (https://github.com/CK7/esom/blob/master/prepare_esom_files.pl) with the 14 read mapping files for each assembly and window and minimum size parameters both set to 3000 bps. Binning itself was done manually for each of the 14 ESOM maps. Overall we recovered 361 bins. Description of the rest of the data will be provided elsewhere.

Melainabacteria BJ4A: Data assembly and genome binning was carried out as described in Hernsdorf et al. [7].

Melainabacteria AS1A-AS3B: Reads for each of the arsenate gut samples were trimmed and assembled using the same method as Margulisbacteria RA1A (see above). Samples were binned using DASTool v1.0 [13] with input bins generated using CONCOCT v0.4.1 [14], ABAWACA v1.00 (https://github.com/CK7/abawaca), MaxBin v2.2 [15], and the ggKbase binning interface (https://ggkbase.berkeley.edu/). For AS1A and AS2A, GC content, coverage, and phylogenetic profile were used for refinement of the genomes.

Curation of genomes derived from metagenomes

Margulisbacteria RA1A: The ESOM bin included 147 scaffolds. The bin was then manually curated by using a mini-assembly approach as described in Sharon et al. [9]. The mini-assembly process is iterative and was done manually as follows. First, the reads from the aac11 sample are mapped to the bin scaffolds using bowtie with parameters as described above. Next, for each scaffold end, all reads that align to the end are collected along with their pair-end mates. These reads are then assembled and the scaffold is elongated based on this local assembly. Elongation process stops once either one or more other scaffolds are linked to the end or when no local assembly can be generated, usually due to low or no coverage. The process is repeated until no further elongation can be done to any of the scaffolds. 23 mini-assembly iterations were performed overall. As was discovered during the process, the high number of scaffolds is due to the presence of close to 80 transposase and integrase genes in the genome. Additionally, we used taxonomic assignment of each scaffold in ggKbase (http://www.ggkbase.berkeley.edu) to evaluate whether scaffolds with only a few genes should be included. Taxonomic assignment to scaffolds works as follows. First, all proteins from all scaffolds were aligned against a comprehensive in-house database of proteins from reference genomes including isolates from public databases (NCBI), high-quality published MAGs (NCBI, IMG), and unpublished MAGs reconstructed in our laboratory. For each protein we identified the best hit and assigned it a species-level taxonomy based on this best hit. Next, we used a majority-voting approach to assign a taxonomy to a scaffold, starting at the domain level all the way to the species level (if possible). This process ends once we reach a level in which no "winner" taxon has more than 50% of the proteins assigned to it. The level above this one provides our taxonomy assignment for the scaffold. Resulting number of scaffolds is 111, with a total length of 3.43 mbp.

Assembly errors were identified and repaired using a previously described method (https://github.com/christophertbrown/fix_assembly_errors/releases/tag/2.00) [1]. Briefly, errors were identified as regions of no coverage by stringently mapped paired-read sequences. Stringent mapping was conducted by first mapping reads to the assembly using Bowtie2 [16] with default parameters for paired-reads. The software repairs assembly errors using Velvet [17], to re-assemble reads that map to those regions based on less-stringent criteria (only one read in the pair has to map to a particular region with less than two mismatches). The final assembly was visually inspected with mapped paired read sequences

using Geneious [18]. As a result of this process, about 98% of the genes remained unchanged. Contigs whose phylogenetic affiliation did not match that of the majority were removed from the genome.

Melainabacteria BJ4A: Local assembly errors were manually identified and repaired and the genome was curated in Geneious. Paired reads were used to fill gaps and to extend and join scaffolds.

Melainabacteria AS1A-AS3B: Local assembly errors were identified and repaired using the previously described method [1].

Single-cell genomics

The generation, identification, sequencing and assembly of single amplified genomes (SAGs) was performed at the Bigelow Laboratory Single Cell Genomics Center (scgc.bigelow.org). Samples were initially passed through a 70 µm (AAA071) or 40 µm (others) strainer (Becton Dickinson) and incubated for 10–60 min with LysoTracker Green (AAA071) or SYTO-9 (others) stains (Thermo Fisher Scientific). Fluorescence-activated cell sorting (FACS) was performed with the BD MoFlo (AAA071) or InFlux Mariner (others) flow cytometers equipped with 488 nm lasers and 70 µm nozzle orifices (Becton Dickinson). Cytometers were triggered to sort on side scatter, and the "single-1 drop" mode was used for purity. For each sample, individual cells were deposited into 384-well plates containing 600 nL per well of 1x TE buffer and stored at −80°C until further processing: 315 or 317 wells were dedicated for single particles, 66 or 64 wells were used as negative controls (no droplet deposition), and 3 wells received 10 particles each to serve as positive controls. The DNA for each cell was amplified using MDA (AAA071) or WGA-X (others), as previously described in Stepanauskas et al. [19]. 16S RNA genes were analyzed as in in Stepanauskas et al. (2017). Shotgun Illumina libraries were created and sequenced, and the reads were assembled using SPAdes v3.9.0 [20] with modifications, as previously described (Stepanauskas et al., 2017). Three bacterial benchmark cultures were used to evaluate SAGs for assembly errors. Benchmark cultures have diverse genome complexity and %GC, resulting in 60% average genome recovery, no non-target and undefined bases, and average frequencies of misassemblies, indels and mismatches per 100 kbp: 1.5, 3.0, and 5.0 [19]. Bacterial symbiont contigs of the protist SAG AAA071-K20 were identified and separated from the host genome using tetramer principal component analysis, relying on the first two principal components [21]. Final SAG assemblies are deposited in the Joint Genome Institute Integrated Microbial Genomic database (https://img.jgi.doe.gov/).

Supplementary Note 1: Central metabolism in Margulisbacteria

Based upon the absence of CO_2 fixation pathways in the genomes of organisms from both environments we anticipate a heterotrophic lifestyle. Based on the more complete aquifer genomes, Riflemargulisbacteria can break down cellulose (cellulose and cellobiose), hemicellulose (mannan, xylo-oligosaccharide, xylo-oligosaccharide, and manno-oligosaccharide), chitin (chitosan), pectin (pectin and pectate), starch (starch, maltose, and limit dextrin), other polysaccharides such as pullulan, and amino sugars such as melibiose and peptidoglycan. These compounds can be fed into a glycolytic pathway. Interestingly, for the transformation of 3-phosphoglyceraldehyde to 1,3-biphosphoglycerate, Margulisbacteria RA1A encodes two different enzymes: glyceraldehyde 3-phosphate dehydrogenase (GAPDH, K00134) and glyceraldehyde ferredoxin oxidoreductase (GAPOR, K11389).

We predict that Margulisbacteria RA1A has a flexible metabolism that can make use of carbohydrates when they are available, and also take up short chain fatty acids and use them to produce carbon storage compounds. Under high carbohydrate availability, Margulisbacteria RA1A could oxidize pyruvate produced by glycolysis to acetyl-CoA by pyruvate:ferredoxin oxidoreductase (PFOR, EC 1.2.7.1). Some acetyl-CoA could then be converted to acetate by phosphotransacetylase and acetate kinase, with production of ATP (**Fig. 3**). Alternatively, pyruvate formed via glycolysis could be

fermented to lactate by one of two lactate dehydrogenases (K00016 or K03778), producing NADH, and the lactate excreted from the cell.

Under low carbohydrate availability, lactate dehydrogenases (K00016) may function in reverse as part of an electron bifurcating complex that includes EtfAB (as described by [22]). In this reaction, lactate taken up from the environment could be oxidized to pyruvate, with simultaneous oxidation of reduced ferredoxin and NAD^+ reduction. The pyruvate could then be fed into the gluconeogenesis pathway to produce starch/glycogen or trehalose. However, this is unlikely to happen in Margulisbacteria RA1A. Alternatively, pyruvate could be transformed to oxaloacetate and used to synthesize amino acids. Similarly, Margulisbacteria RA1A could take up ethanol from the environment and use alcohol dehydrogenase (EC 1.1.1.1) encoded by *yiaY* (K013954) or *adh* (K00001, iron-containing) to convert it to acetaldehyde, which can be transformed to acetyl-CoA by acetaldehyde ferredoxin oxidoreductase (AFOR, K03738). Acetyl-CoA could be fed into a pathway for synthesis of fatty acids. Margulisbacteria AA1A seems to use acetate as a source of acetyl-CoA in a reaction catalyzed by NAD^+-dependent acetyl-CoA synthetase (K01895). This enzyme was not identified in Margulisbacteria RA1A (Supplementary Data 2).

The five TCA cycle enzymes identified in Margulisbacteria RA1A produce aspartate and glutamate (the precursors to eight other amino acids), lysine, methionine and tetrapyroles (the prosthetic group in cytochromes and chlorophyll). The conversion of alpha-ketoglutarate to succinyl-CoA is carried out by a heterodimeric (KorAB) 2-oxoglutarate:ferredoxin oxidoreductase (OFOR, EC 1.2.7.3) in the organisms from both environments. This is notable because it is a ferredoxin-based mechanism, in place of the more widely used alpha-ketoglutarate dehydrogenase, which uses NAD^+. Malic enzyme (K00027) may convert malate into pyruvate and CO_2 or decarboxylate oxaloacetate into pyruvate that is required for synthesis of aspartate. Margulisbacteria AA1A seems to have all the enzymes of the TCA cycle, including alpha-ketoglutarate dehydrogenase. In Margulisbacteria AA1A, pyruvate can also be converted to oxaloacetate by pyruvate carboxylase (K01958). In addition, a full shikimate biosynthetic pathway that could be the source of aromatic amino acids or folate was identified. Amino acids for which synthesis pathways were not identified (Supplementary Data 3) could be transported inside the cell by a putative oligopeptide ABC and digested by one of multiple peptidases. For all Margulisbacteria studied here, enzymes of the oxidative decarboxylation part of the pentose phosphate pathway (PPP) are absent. The only PPP enzymes in the genomes appear to be those involved in formation of precursors of ribose and deoxyribose (**Fig. 3**).

Supplementary Note 2: An independent lifestyle seems to be common in Riflemargulisbacteria

Consistent with a largely independent lifestyle for all Riflemargulisbacteria studied here, adenine and guanosine ribonucleotides can be synthesized, as well as uridine monophosphate (the precursor of pyrimidine ribonucleotide UTP). UTP could be converted to pyrimidine deoxyribonucleotide (dCTP), but key enzymes for the synthesis of dTTP could not be found. The genomes have pathways for the production of cofactors and vitamins, as well as biosynthesis of electron carriers. Specifically, we predict the production of riboflavin, NAD, coenzyme A, tetrahydrofolate, heme (precursor of cytochromes), and the following vitamins: thiamine (Vit B1), pantothenate (Vit B5, precursor of coenzyme A), biotin (Vit B7), pyridoxal (a form of Vit B6), and pimeloyl-ACP (precursor of biotin). Vitamin B12 could be transported into the cell. Margulisbacteria also have the ability to synthesize fatty acids (initiation and elongation), glycerolipids and C5 isoprenoids via the 2-C-methyl-D-erythritol 4-phosphate/1-deoxy-D-xylulose 5-phosphate (MEP/DOXP) pathway (Supplementary Data 3). Unlike Margulisbacteria RA1A, Margulisbacteria AA1A may contain a full pathway for assimilatory sulfate reduction.

For all Margulisbacteria we identified genes involved in lipopolysaccharide biosynthesis that would be indicative of a Gram-negative cell wall, a characteristic also predicted for Melainabacteria and Cyanobacteria. However, genes for 10 or 12 of the 12 steps in lipid A biosynthesis were not identified. Supporting Gram-negative status is the presence of genes for colanic acid biosynthesis, porins, and TonB transporters. Like Melainabacteria, Margulisbacteria are motile by polar flagella. Both groups possess type IV pilus assembly proteins (Supplementary Data 2). Additionally, Margulisbacteria RA1A have twitching motility, which seems to be absent in the ocean organisms. Twitching motility may be a capacity only relevant for life in association with solid surfaces and not needed for planktonic ocean-dwelling bacteria. Margulisbacteria RA1A also has chemotaxis genes and response regulators that would enable motility triggered by external stimuli.

Supplementary Note 3: Cytoplasmic hydrogenases in Riflemargulisbacteria

Cytoplasmic NiFe hydrogenases are reversible enzymes, and in Riflemargulisbacteria groups 3b, 3c, and 3d NiFe hydrogenases may be involved in reoxidation of reduced electron carriers from central metabolic pathways. Cyanobacteria also have cytosolic NiFe hydrogenases, a group 3d and a group 2a. The uptake hydrogenase in group 2a (encoded by *hupS* and *hupL*) [23] is absent in Riflemargulisbacteria and related lineages, and it is thought to have evolved more recently to harvest the H_2 generated during N_2-fixation in N_2-fixing Cyanobacteria [24]. Margulisbacteria represented by genome GW2A is the only organism predicted to encode a cytoplasmic NAD(P)-linked group 3d NiFe hydrogenase (HoxEFUYH) (**Fig. 4a**), the other type found in Cyanobacteria (*e.g., Synechocystis spp.*). In general, Hox hydrogenases are thought to use NAD(P)H to reduce H^+, however this hydrogenase accepts electrons from flavodoxin/ferredoxin during pyruvate fermentation involving NifJ and PFOR [25]. Thus, Hox rather than nitrogenase may reoxidize ferredoxin in Margulisbacteria GW2A.

Another kind of cytoplasmic hydrogenase identified in Riflemargulisbacteria is a group 3b NADP-coupled NiFe hydrogenase. This hydrogenase in Margulisbacteria RA1A clusters phylogenetically with other enzymes that have not been described experimentally (**Fig. 4a**). These soluble hydrogenases generate H_2 (or hydrogen sulfide when polysulfide is available in the archaeon *Pyrococcus furiosus*) to dispose of excess reducing equivalents. Reduced nicotinamide adenine dinucleotide phosphate (NADPH) is inferred to be the electron donor for this hydrogenase [26]. Enzymes such as isocitrate dehydrogenase may be the source of NADPH. Under certain conditions, it could also be used in the reverse direction to save energy for anabolism in the form of NADPH [27]. Flanking the Margulisbacteria RA1A genes encoding the NADP-reducing NiFe hydrogenase we identified genes encoding ferredoxin (K05337) and a pyruvate-ferredoxin/flavodoxin oxidoreductase (K03737, *por/nifJ*) that generates reduced ferredoxin during conversion of pyruvate to acetyl-coA (with the concomitant release of CO_2). This enzyme has a similar function to PFOR, which is implicated in the transformation of pyruvate to acetyl-CoA (see above). However, reduced ferredoxin formed by either enzyme is not an effective electron donor for the NADP-reducing NiFe hydrogenase [28], so its re-oxidation must be achieved by another enzyme. In *Klebsiella pneumoniae* a flavodoxin (NifF) involved in the reaction catalyzed by NifJ acts as an electron carrier [29] to nitrogenase, but a gene encoding flavodoxin was not identified. In Margulisbacteria RA1A, reduced ferredoxin may be the electron donor for the nitrogenase.

Among the sister clades of Cyanobacteria, Riflemargulisbacteria is the only one that possess a group 3c NiFe methyl viologen-reducing (Mvh) NiFe hydrogenase, an ancestral enzyme known to oxidize H_2 or formate and transfer electrons to the heterodisulfide reductase subunit A (HdrA). Two kinds of cytoplasmic Group 3c NiFe hydrogenase were identified (**Fig. 4a**). We also identified HdrA, which normally bifurcates electrons to the other heterodisulfide reductase subunits (HdrB and HdrC) and ferredoxin in methanogens that lack cytochromes, or to formylmethanofuran dehydrogenase [30, 31].

However, Margulisbacteria RA1A does not encode HdrB, HdrC or formylmethanofuran dehydrogenase, so HdrA is inferred to function in another context (**Fig. 3**). A similar gene arrangement has been found in candidate phyla Zixibacteria from the same ecosystem [32].

Supplementary Note 4: Putative formate dehydrogenase-Ehr complex in Riflemargulisbacteria and Saganbacteria

In Margulisbacteria RA1A, the predicted Ehr complex includes homologs to NADH dehydrogenase subunits NuoB and NuoD. Notably, the gene encoding NuoD also encodes a homolog of NuoC, and the gene encoding NuoB encodes a homolog of NuoI, similar to Ehr complexes in Firmicutes and hyperthermophilic bacteria and archaea [33]. Additionally, two homologs to multiple resistance and pH adaptation (Mrp) antiporter-like subunits (annotated as HyfB and HyfF), a homolog to the quinone binding subunit (NuoH) of NADH:Ubiquinone oxidoreductase (Nuo), and a transmembrane subunit of *E. coli* hydrogenase 4 (HyfE) are comprised by this complex.

The gene encoding the catalytic subunit of the molybdenum/tungsten-dependent formate dehydrogenase (FdhA) was found next to genes annotated as the Nuo electron transfer subunits NuoE and NuoF, in addition to a thioredoxin-like protein (Supplementary Fig. 3b). Together, these four proteins may constitute the formate dehydrogenase partner of the Ehr complex. The putative formate dehydrogenase in Riflemargulisbacteria resembles the subunit composition of the molybdenum-dependent enzyme in *Ralstonia eutropha* [34]. In *R. eutropha,* the formate dehydrogenase is comprised of four subunits with the following domain composition: the alpha subunit (molybdopterin oxidoreductase and molybdopterin dinucleotide binding domains) harbors three 4Fe-4S plus two 2Fe-2S cluster binding domains in the N-terminus (resembling NuoG); the beta subunit contains binding domains for one 4Fe-4S cluster, a flavin mononucleotide (FMN), and a nicotinamide adenine dinucleotide (NAD^+) (similar to NuoF); the gamma subunit contains a binding domain for a 2Fe-2S cluster (similar to NuoE); and the delta subunit may only have a structural role because it does not seem to be involved in electron transfers [34, 35, 36].

The enzyme in Margulisbacteria RA1A (and Saganbacteria RX5A) seems to comprise four subunits as well, but it is different from the enzyme in *R. eutropha* in that it contains only one 2Fe-2S cluster-binding domain in the alpha subunit (as opposed to three), an additional binding domain for a 4Fe-4S cluster in the beta subunit (as opposed to one), and a 2Fe-2S cluster-binding domain in the delta subunit. Also, no NAD^+-binding domain was observed in the beta subunit, although it was predicted to be present for Saganbacteria RX3A and RX7A, in addition to an alpha-helical ferredoxin domain (2 x 4Fe-4S cluster). In the genomic region encoding FdhA in Margulisbacteria RA1A we also identified genes encoding a transporter for molybdate/tungstate required for the incorporation of a molybdenum or tungstate cofactor in formate dehydrogenase and, FdhD a protein known to activate FdhA. Similar genomic regions in Saganbacteria differ from those in Margulisbacteria due to the presence of genes encoding a redox sensing transcriptional repressor (*rex*); *oxlT*, a phosphate selective porin (*oprO*), and additional genes involved in molybdenum cofactor biosynthesis (Supplementary Fig. 3b).

The possibility that Margulisbacteria RA1A has a formate dehydrogenase raises the question of the source of formate. In *E. coli* growing fermentatively, formate may be produced by pyruvate formate lyase [37]. A gene encoding pyruvate formate-lyase was not found in Margulisbacteria RA1A. However, a gene encoding an oxalate:formate antiporter (*oxlT;* K08177) is present in this genome and we predict that it could be used to import formate into the cell.

FdhA was predicted to be encoded by multiple lineages sibling to the Cyanobacteria. Phylogenetically, the FdhA in Margulisbacteria RA1A and Saganbacteria RX3A is related to a protein found in Modulibacteria (Candidate Phylum KSB3). However, FdhA in Saganbacteria RX4A and

Melainabacteria RX6A cluster with those in *Syntrophobacter fumaroxidans*, an organism that has been shown to catalyze CO_2 reduction to formate [36, 38]. Saganbacteria RX5A and RX7A form a cluster on their own, whereas FdhA in Melainabacteria BJ4A clusters with that in Mycobacteria; and FdhA in Sericytochromatia LSPB_72 clusters with those in Cyanobacteria.

Supplementary Note 5: Enigmatic hydrogenases in Saganbacteria

In the genomic region of the enigmatic group 4 hydrogenase found in Saganbacteria RX5A we identified a gene encoding a putative molybdopterin-containing reductase. The predicted protein was annotated as the catalytic subunit of a formate dehydrogenase (FdfH or alternatively FdoG) by HMMs, but it is also similar to the catalytic subunit of a periplasmic nitrate oxidoreductase (NapA), although it lacks key residues for nitrate binding. This protein is predicted to have only one 4Fe-4S cluster-binding domain (instead of three 4Fe-4S cluster-binding domains like the FdhA in Margulisbacteria RA1A) in addition to the molybdopterin oxidoreductase- and molybdopterin-binding domains. In this genomic region, adjacent to *fdfH* there are other genes (*e.g., asrA-like, asrB-like, cooF-like*) that may play similar roles than *nuoE-like* and *nuoF-like* in Margulisbacteria RA1A. Genes encoding putative anaerobic sulfite reductase subunits AsrA and AsrB are predicted to be involved in electron transfer. For instance, the predicted AsrA-like protein contains an alpha-helical ferredoxin (2 x 4Fe-4S cluster-binding domain). The predicted AsrB-like protein includes a ferredoxin reductase-type FAD-binding domain, an oxidoreductase FAD/NAD-binding domain, and dihydroorotate dehydrogenase Fe-S cluster-binding domains. The CooF-like protein was predicted to include binding domains for at least two 4Fe-4S clusters. Together, these subunits may constitute a single enzyme or the equivalent of an oxidoreductase module in the Mbh-type hydrogenases.

Other subunits encoded in this complex in Saganbacteria RX5A include the small and large subunits of the group 4 NiFe hydrogenase (homologs of NuoB and NuoD, respectively), homologs of NuoI (2 x 4Fe-4S cluster-binding domains), NuoC, and NuoH; two membrane-bound subunits, and two Mrp-antiporters (antiporter module) (Supplementary figure S3e). Therefore, this complex is most likely an Mbh hydrogenase type enzyme like the ones observed in other bacteria and archaea [39].

The confirmed group 4 NiFe hydrogenase in RX6A comprises homologs of NuoI (2 x 4Fe-4S cluster-binding domains), NuoC, and NuoH; three membrane-bound subunits (homologs of NuoK and NuoL), and three Mrp-antiporters subunits (Supplementary figure S3f). The other putative group 4 NiFe hydrogenase or hydrogenase-related complex in this genome includes a protein with a predicted molybdopterin oxidoreductase domain (but no Fe-S cluster-binding domains) that could not be confirmed by phylogeny as a formate dehydrogenase. However, genes encoding AsrA- and AsrB-like proteins are also present in this region, as well as a gene encoding a cooF-like protein (Supplementary figure S3d) with predicted domain composition similar to the proteins in the RX5A complex.

Lastly, Saganbacteria RX4A encodes a complex comprising FdhA and Asr-like proteins that resembles the formate dehydrogenases found in Saganbacteria RX5A and Margulisbacteria RA1A (Supplementary figure S3c). Given that this genome also encodes an Ehr complex, it is possible that this oxidoreductase couples its action mechanism with that of the Ehr, like the putative formate dehydrogenase-Ehr complex proposed for Margulisbacteria.

Supplementary Note 6: Cytoplasmic hydrogenases and electron transport chain configurations in Melainabacteria

It was not possible to assign the FeFe hydrogenases in Melainabacteria to a specific taxonomic group (e.g., glutamate synthase-linked (group A2); electron bifurcating (group A3); or formate dehydrogenase-linked (A4); Supplementary Fig. 4). Nevertheless, all the FeFe hydrogenases in

Melainabacteria are trimeric (encoded by gene homologs of *hydABC)* and have predicted protein domains that resemble group 2 (G2) bifurcating hydrogenases [40]. They also display two kinds of modular structure [41] in their catalytic subunit (HydA): one kind is predicted to display a modular structure type 3c like that of Melainabacteria RX6A (and Margulisbacteria RA1A); and the other kind is predicted to be type 3a. This type was predicted for the second FeFe hydrogenase in Melainabacteria RX6A, and the FeFe hydrogenase in Melainabacteria AS2A, GW8A, and GW9A. GW9A has two of these FeFe hydrogenases, although they differ in the domain structure of the HydB subunit. In many cases, we also noticed the presence of genes encoding histidine kinases next to the genes encoding the FeFe hydrogenases. These genes have been suggested to be involved in posttranslational modifications to regulate the activity of the bifurcating enzymes [40].

Melainabacteria BJ4A has genes (*cydA*, K00425 and *cydB*, K00426) encoding a complete cytochrome *d* ubiquinol oxidase (complex IV). Additionally, Melainabacteria BJ4A has another gene encoding cytochrome *d* ubiquinol oxidase subunit I (*cydA*) directly upstream from a second set of cytochrome b_6 genes. Melainabacteria LO5A also encodes a cytochrocome *d* ubiquinol oxidase subunit I (*cydA*), but no cytochrome b_6 gene was found in the vicinity (Supplementary Data 22).

Melainabacteria BJ4A and HO7A also contain type C heme-copper O_2 reductase genes directly downstream from one set of genes of the cytochrome b_6f complex (*petB, petB, petC*). In Melainabacteria BJ4A, the O_2 reductase is partly encoded as a gene fusion, whereas Melainabacteria HO7A only has two genes (*ccoN and ccoO*) (Supplementary Data 22).

We predict that the genes encoding a cytoplasmic nitrate/nitrite oxidoreductase (NXR; **Fig. 5**) and cytochrome b_6 (*petB*), which occur in the same genomic region in Melainabacteria LO5A, are involved in nitrate/nitrite reduction. A potentially similar gene arrangement was reported in other Melainabacteria, where genes were predicted to encode a cytochrome *bc*-related protein and a nitrate reductase (NarG) [42].

Supplementary Note 7: Potentially interesting genes shared with Cyanobacteria

From the perspective of the distribution of genes shared with Cyanobacteria, Riflemargulisbacteria genomes encode circadian clock proteins KaiC and KaiB also found in some Melainabacteria. Margulisbacteria also possess *bchE*, a gene encoding anaerobic magnesium-protoporphyrin IX monomethyl ester cyclase (chlorophyll biosynthesis), which is also found in phototrophic bacteria such as Chloroflexi, Chlorobi, Heliobacillus, and purple bacteria [43]. Only *kaiC* was found in the single cell genomes of the ocean organisms.

Supplementary Note 8: Other interesting findings

Genes that encode multicomponent Na^+:H^+ antiporter complexes were identified in Marinamargulisbacteria genomes, but not in the Riflemargulisbacteria genomes (Supplementary Data 2). The presence of this complex may be an adaptation to life in the ocean vs. freshwater. However, Margulisbacteria, Saganbacteria and Melainabacteria genomes encode K^+-stimulated pyrophosphate-energized Na^+ pumps that may also pump H^+ [44]. This pyrophosphatase may help maintain a low internal Na^+ concentration by working in parallel with a Na^+/H^+ antiporter [45].

A key enzyme of the Entner-Doudoroff (ED) pathway, (2-dehydro-3-deoxyphosphogluconate aldolase/(4S)-4-hydroxy-2-oxoglutarate aldolase; EC 4.1.2.14, 4.1.3.42) was identified in Riflemargulisbacteria, and a few of the Saganbacteria and Melainabacteria studied here (Supplementary Data 2). In all cases, its function is uncertain, because the rest of the pathway seems to be missing. Due to the low ATP yields associated with sugars degradation through the ED pathway it is not commonly found among anaerobic bacteria.

In terms of defense, we identified genes including arsenite methyltransferase (As resistance), superoxide dismutase (oxidative stress), nitric oxide reductase (protects 4Fe-4S NO-sensitive enzymes) and Type I CRISPR-Cas systems (phage defense) (Supplementary Data 2).

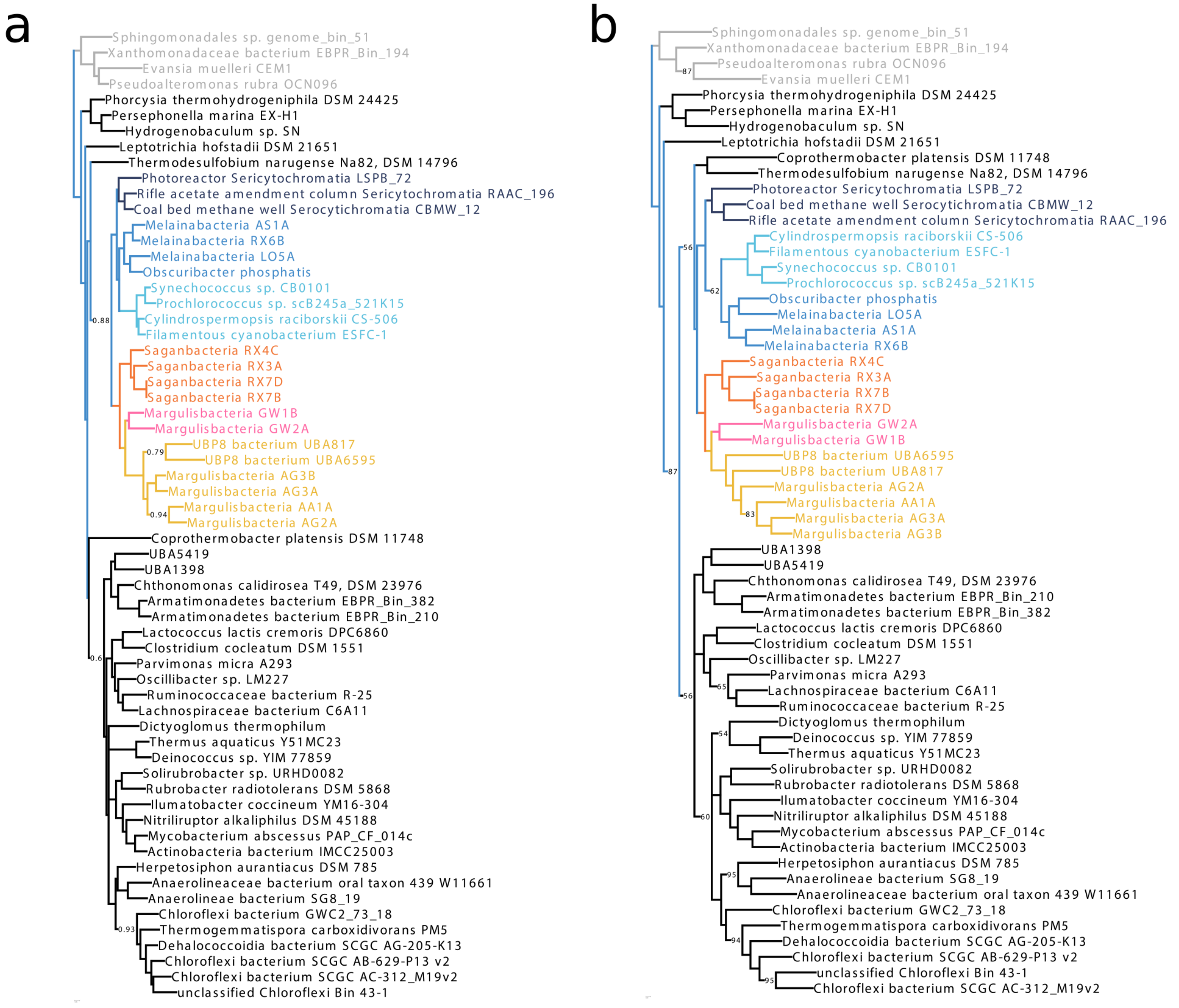

Supplementary Fig. 1. Detailed concatenated 56 marker proteins phylogenetic tree. Inferred with **a.** Phylobayes CAT + GTR and **b.** IQ-tree.

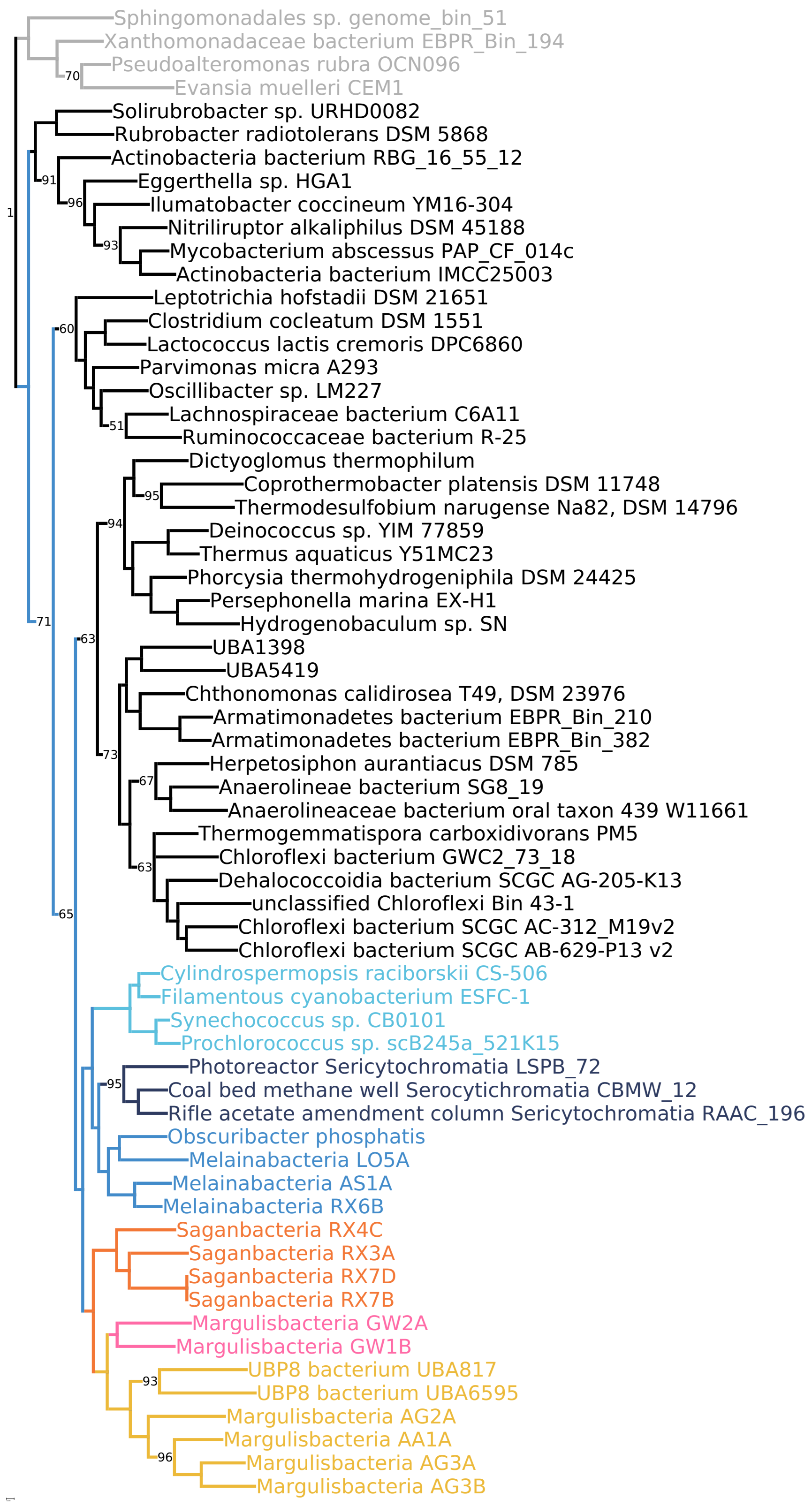

Supplementary Fig. 2. Detailed concatenated 16 ribosomal proteins IQ-TREE phylogenetic tree.

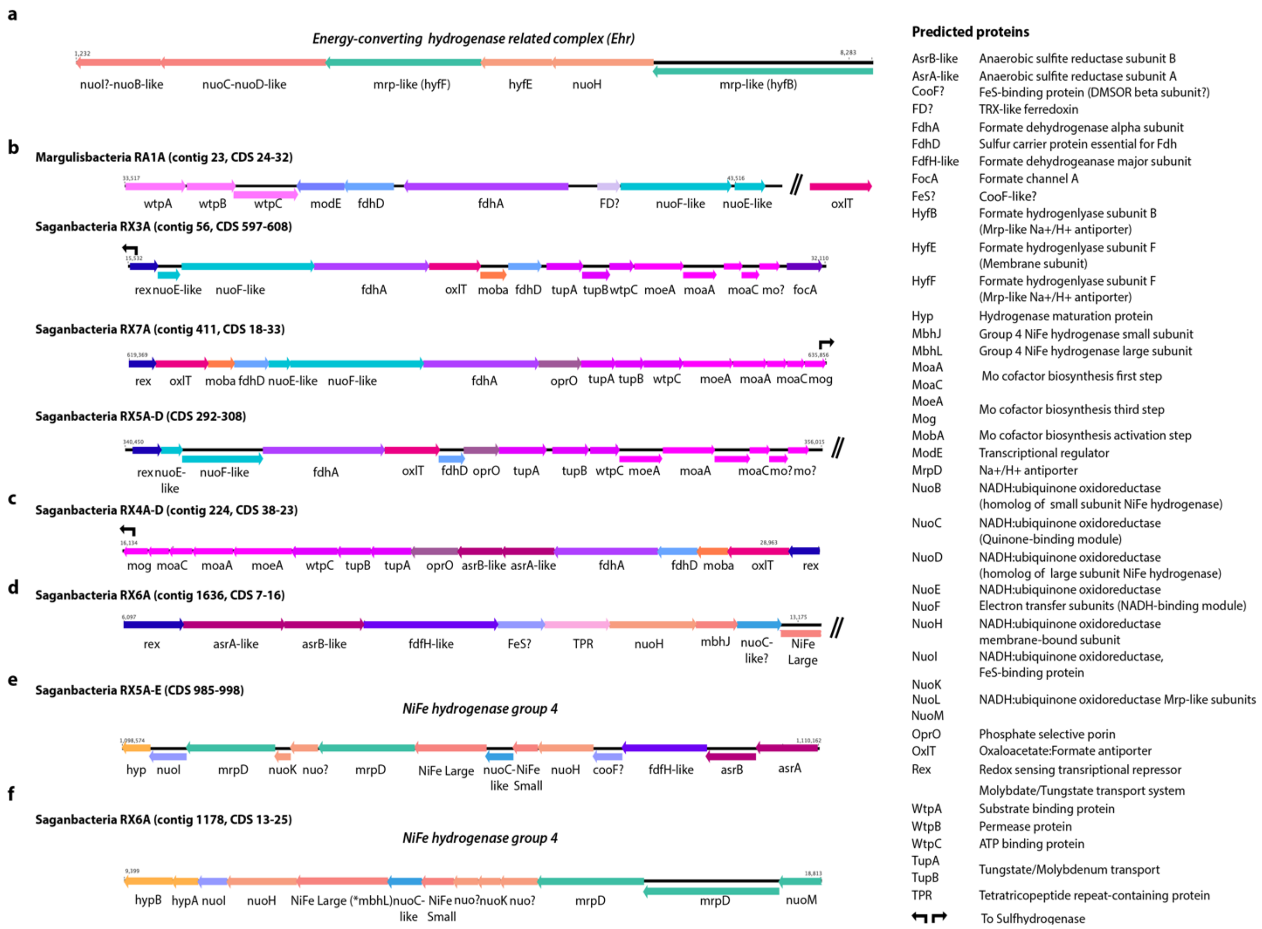

Supplementary Fig. 3. Genomic regions encoding key protein complexes. a. Genes encoding the Ehr complex in Margulisbacteria RA1A include homologs to the small and large subunits of group 4 NiFe hydrogenase (without the catalytic residues) followed by genes encoding antiporter-like and membrane-bound subunits. A similar gene arrangement was found in all the genomes that harbor this kind of hydrogenase. **b.** Genomic region in Margulisbacteria RA1A, Saganbacteria RX3A, Saganbacteria RX7A, and Saganbacteria RX5A-D encoding molybdopterin-binding reductases identified as formate dehydrogenase (FdhA; **Fig. 5**). **c.** Genomic region in Saganbacteria RX4A-D encoding a formate dehydrogenase, and proteins similar to an anaerobic sulfite reductase. **d.** Genomic region in Saganbacteria RX6A encoding a formate dehydrogenase, proteins similar to an anaerobic sulfite reductase, and a partial NiFe hydrogenase that could not be identified. **e.** Genomic region in Saganbacteria RX5A-E encoding a formate dehydrogenase, proteins similar to an anaerobic sulfite reductase, and membrane-bound NiFe hydrogenase with antiporter-like and membrane-bound subunits (**Fig. 4b**, Mrp-Mbh-type). **f.** Genomic region in Saganbacteria RX6A containing genes encoding a membrane-bound NiFe hydrogenase with antiporter-like subunits (**Fig. 4b**, Mrp-Mbh-type).

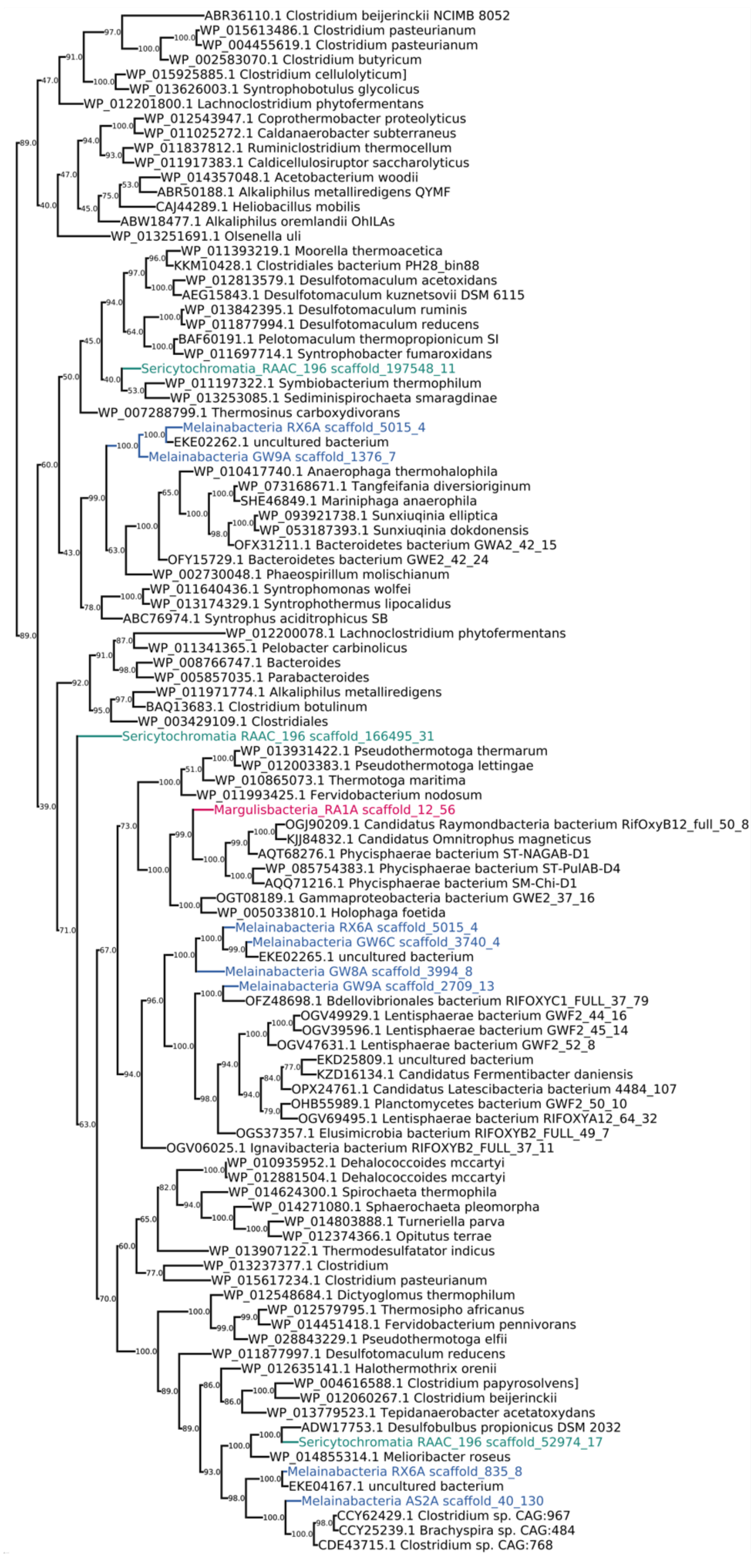

Supplementary Fig. 4. Phylogenetic tree of the catalytic subunit of FeFe hydrogenases. Bayesian phylogeny indicating the positions of Margulisbacteria (pink), Melainabacteria (blue), and Sericytochromatia (green) FeFe hydrogenase catalytic subunits. Scale bar indicates substitutions per site. Branches with a posterior support of below 0.5 were collapsed.

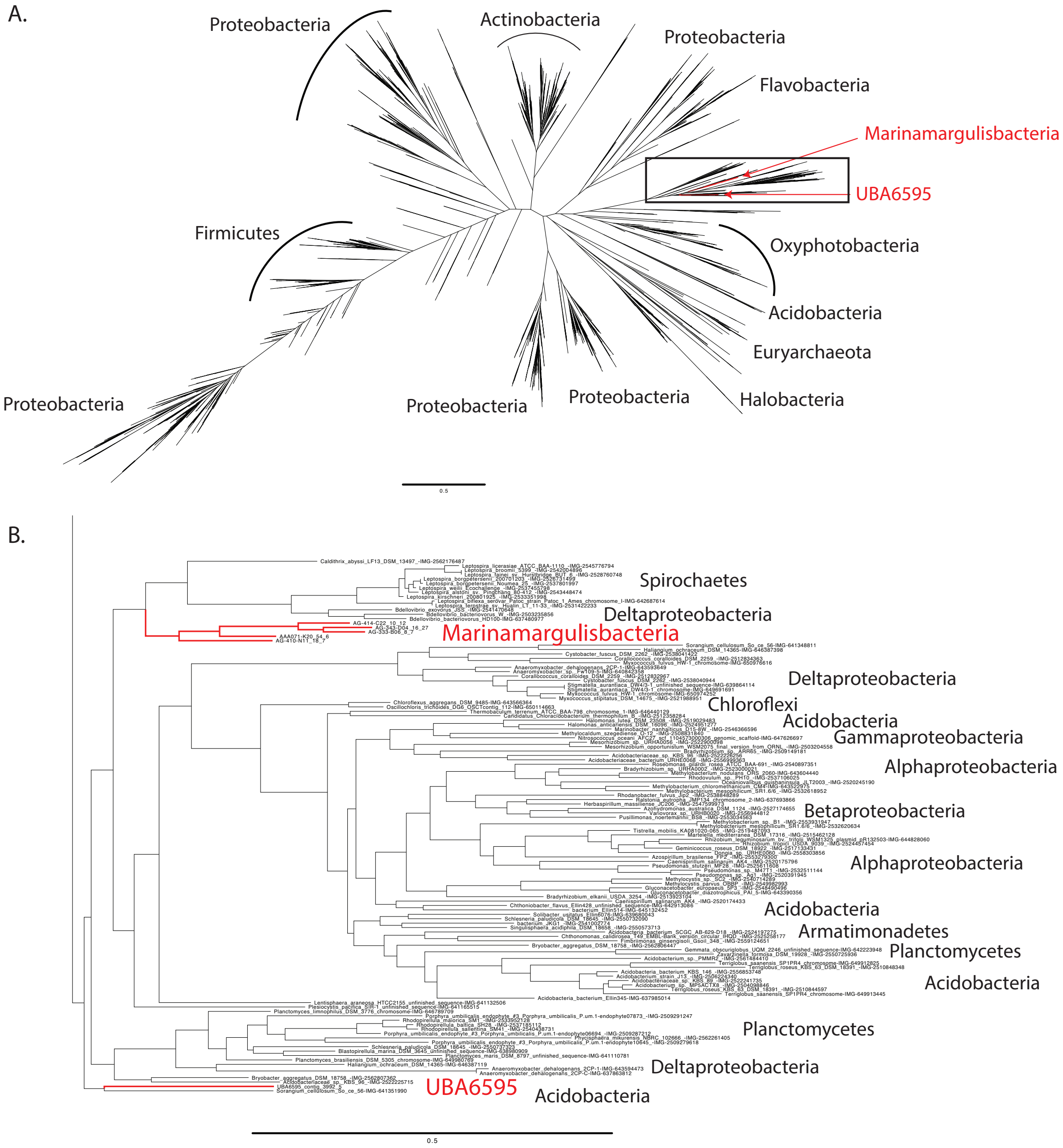

Supplementary Fig. 5. Phylogenetic tree of type A heme-copper oxygen reductases. a. Maximum-likelihood tree of CoxA (cytochrome *c* oxidase subunit I). Branches from Marinamargulisbacteria are highlighted in red. Box highlights the clade containing Marinamargulisbateria as well as a number of species from different phyla. **b.** Zoom in of the clade containing Marinamargulisbacteria (highlighted in red). Various phyla are represented in this clade, likely as a result of rampant horizontal gene transfer.

Supplementary References

1. Brown CT*, et al.* Unusual biology across a group comprising more than 15% of domain Bacteria. *Nature* **523**, 208-211 (2015).

2. Anantharaman K*, et al.* Thousands of microbial genomes shed light on interconnected biogeochemical processes in an aquifer system. *Nat Commun* **7**, https://doi.org/10.1038/ncomms13219 (2016).

3. Handley KM*, et al.* Disturbed subsurface microbial communities follow equivalent trajectories despite different structural starting points. *Environ Microbiol* **17**, 622-636 (2015).

4. Wrighton KC*, et al.* Fermentation, hydrogen, and sulfur metabolism in multiple uncultivated bacterial phyla. *Science* **337**, 1661-1665 (2012).

5. Martinez-Garcia M*, et al.* Unveiling *in situ* interactions between marine protists and bacteria through single cell sequencing. *ISME J* **6**, 703-707 (2012).

6. Ino K*, et al.* Ecological and genomic profiling of anaerobic methane-oxidizing archaea in a deep granitic environment. *ISME J* **12**, 31 (2017).

7. Hernsdorf AW*, et al.* Potential for microbial H_2 and metal transformations associated with novel bacteria and archaea in deep terrestrial subsurface sediments. *ISME J* **11**, 1915-1929 (2017).

8. Peng Y, Leung HCM, Yiu SM, Chin FYL. IDBA-UD: a de novo assembler for single-cell and metagenomic sequencing data with highly uneven depth. *Bioinformatics* **28**, 1420-1428 (2012).

9. Sharon I, Morowitz MJ, Thomas BC, Costello EK, Relman DA, Banfield JF. Time series community genomics analysis reveals rapid shifts in bacterial species, strains, and phage during infant gut colonization. *Genome Res* **23**, 111-120 (2013).

10. Ultsch A, Mörchen F. *ESOM-Maps: tools for clustering, visualization, and classification with Emergent SOM*. Univ. (2005).

11. Dick GJ*, et al.* Community-wide analysis of microbial genome sequence signatures. *Genome biol* **10**, 1-16 (2009).

12. Langmead B, Trapnell C, Pop M, Salzberg SL. Ultrafast and memory-efficient alignment of short DNA sequences to the human genome. *Genome Biol* **10**, https://doi.org/10.1186/gb-2009-10-3-r25 (2009).

13. Sieber, C. M. K., et al. Recovery of genomes from metagenomes via a dereplication, aggregation and scoring strategy. *Nat Microbiol* **3**, https://doi.org/10.1038/s41564-018-0171-1 (2018).

14. Alneberg J*, et al.* Binning metagenomic contigs by coverage and composition. *Nat Methods* **11**, 1144-1146 (2014).

15. Wu Y-W, Simmons BA, Singer SW. MaxBin 2.0: an automated binning algorithm to recover genomes from multiple metagenomic datasets. *Bioinformatics* **32**, 605-607 (2016).

16. Langmead B, Salzberg SL. Fast gapped-read alignment with Bowtie 2. *Nat Methods* **9**, 357-359 (2012).

17. Zerbino DR, Birney E. Velvet: algorithms for de novo short read assembly using de Bruijn graphs. *Genome Res* **18**, 821-829 (2008).

18. Kearse M*, et al.* Geneious Basic: an integrated and extendable desktop software platform for the organization and analysis of sequence data. *Bioinformatics* **28**, 1647-1649 (2012).

19. Stepanauskas R*, et al.* Improved genome recovery and integrated cell-size analyses of individual uncultured microbial cells and viral particles. *Nat Commun* **8**, https://doi.org/10.1038/s41467-017-00128-z (2017).

20. Bankevich A*, et al.* SPAdes: a new genome assembly algorithm and its applications to single-cell sequencing. *J Comput Biol* **19**, 455-477 (2012).

21. Woyke T*, et al.* Assembling the marine metagenome, one cell at a time. *PLoS ONE* **4**, e5299 (2009).

22. Weghoff MC, Bertsch J, Müller V. A novel mode of lactate metabolism in strictly anaerobic bacteria. *Environ Microbiol* **17**, 670-677 (2015).

23. Appel J. The physiology and functional genomics of cyanobacterial hydrogenases and approaches towards biohydrogen production. In: *Functional genomics and evolution of photosynthetic systems* (eds Burnap R, Vermaas W). Springer, Dordrecht (2012).

24. Boyd ES, Schut GJ, Adams MW, Peters JW. Hydrogen metabolism and the evolution of biological respiration. *Microbe* **9**, 361-367 (2014).

25. Gutekunst K, Chen X, Schreiber K, Kaspar U, Makam S, Appel J. The bidirectional NiFe-hydrogenase in *Synechocystis sp*. PCC 6803 Is reduced by flavodoxin and ferredoxin and Is essential under mixotrophic, nitrate-limiting conditions. *J Biol Chem* **289**, 1930-1937 (2014).

26. Ma K, Adams M. Sulfide dehydrogenase from the hyperthermophilic archaeon *Pyrococcus furiosus*: a new multifunctional enzyme involved in the reduction of elemental sulfur. *J Bacteriol* **176**, 6509-6517 (1994).

27. Silva PJ*, et al.* Enzymes of hydrogen metabolism in *Pyrococcus furiosus*. *Eur J Biochem* **267**, 6541-6551 (2000).

28. Ma K, Zhou HZ, Adams MWW. Hydrogen production from pyruvate by enzymes purified from the hyperthermophilic archaeon, *Pyrococcus furiosus*: a key role for NADPH. *FEMS Microbiol Lett* **122**, 245-250 (1994).

29. Shah VK, Stacey G, Brill WJ. Electron transport to nitrogenase. Purification and characterization of pyruvate: flavodoxin oxidoreductase. The *nifJ* gene product. *J Biol Chem* **258**, 12064-12068 (1983).

30. Vignais PM, Billoud B. Occurrence, classification, and biological function of hydrogenases: an overview. *Chem Rev* **107**, 4206-4272 (2007).

31. Costa KC, Lie TJ, Xia Q, Leigh JA. VhuD facilitates electron flow from H_2 or formate to heterodisulfide reductase in *Methanococcus maripaludis*. *J Bacteriol* **195**, 5160-5165 (2013).

32. Castelle CJ*, et al.* Extraordinary phylogenetic diversity and metabolic versatility in aquifer sediment. *Nat Commun* **4**, https://doi.org/10.1038/ncomms3120 (2013).

33. Marreiros BC, Batista AP, Duarte AM, Pereira MM. A missing link between complex I and group 4 membrane-bound [NiFe] hydrogenases. *Biochim Biophys Acta* **1827**, 198-209 (2013).

34. Oh JI, Bowien B. Dual control by regulatory gene fdsR of the fds operon encoding the NAD^+-linked formate dehydrogenase of *Ralstonia eutropha*. *Mol Microbiol* **34**, 365-376 (1999).

35. Friedebold J, Frank Mayer E, Bill AXT, Bowien B. Structural and immunological studies on the soluble formate dehydrogenase from *Alcaligenes eutrophus*. *Biol Che H-S* **376**, 561-568 (1995).

36. Maia LB, Moura JJ, Moura I. Molybdenum and tungsten-dependent formate dehydrogenases. *J Biol Inorg Chem* **20**, 287-309 (2015).

37. Skibinski DAG*, et al.* Regulation of the hydrogenase-4 operon of *Escherichia coli* by the 54-dependent transcriptional activators FhlA and HyfR. *J Bacteriol* **184**, 6642-6653 (2002).

38. de Bok FA*, et al.* Two W-containing formate dehydrogenases (CO_2-reductases) involved in syntrophic propionate oxidation by *Syntrophobacter fumaroxidans*. *Eur J Biochem* **270**, 2476-2485 (2003).

39. Schut GJ, Boyd ES, Peters JW, Adams MW. The modular respiratory complexes involved in hydrogen and sulfur metabolism by heterotrophic hyperthermophilic archaea and their evolutionary implications. *FEMS Microbiol Rev* **37**, 182-203 (2013).

40. Poudel S*, et al.* Unification of [FeFe]-hydrogenases into three structural and functional groups. *Biochim Biophys Acta* **1860**, 1910-1921 (2016).

41. Calusinska M, Happe T, Joris B, Wilmotte A. The surprising diversity of clostridial hydrogenases: a comparative genomic perspective. *Microbiology* **156**, 1575-1588 (2010).

42. Soo RM, Hemp J, Parks DH, Fischer WW, Hugenholtz P. On the origins of oxygenic photosynthesis and aerobic respiration in Cyanobacteria. *Science* **355**, 1436-1440 (2017).

43. Mulkidjanian AY*, et al.* The cyanobacterial genome core and the origin of photosynthesis. *Proc Nat Acad Sci USA* **103**, 13126-13131 (2006).

44. Baykov AA, Malinen AM, Luoto HH, Lahti R. Pyrophosphate-fueled Na^+ and H^+ transport in prokaryotes. *Microbiol Mol Biol Rev* **77**, 267-276 (2013).

45. Malinen AM, Belogurov GA, Baykov AA, Lahti R. Na^+-pyrophosphatase: a novel primary sodium pump. *Biochemistry* **46**, 8872-8878 (2007).

46. Lolkema JS, Chaban Y, Boekema EJ. Subunit composition, structure, and distribution of bacterial V-type ATPases. *J Bioenerg Biomembr* **35**, 323-335 (2003).

47. Parks DH, Imelfort M, Skennerton CT, Hugenholtz P, Tyson GW. CheckM: assessing the quality of microbial genomes recovered from isolates, single cells, and metagenomes. *Genome Res*, gr. 186072.186114 (2015).
