# Supplementary material for: Hydrogen-based metabolism as an ancestral trait in lineages sibling to the Cyanobacteria
Source: Nat Commun. 2019 Jan 28;10:463. doi: 10.1038/s41467-018-08246-y (PMC6349859; doi:10.1038/s41467-018-08246-y)
Supplement: Supplementary file 3 — Description of Additional Supplementary Files [file 41467_2018_8246_MOESM3_ESM.pdf]

Description of Additional Supplementary Files

File Name: Supplementary Data 1
Description: General genome information. Genomes with ANI 95.0 - 99.0% are shaded in gray. The representative genome for a given cluster is listed first in bold.

File Name: Supplementary Data 2
Description: Functional orthologs involved in central metabolism predicted to be encoded in the studied genomes by HMMs of KO. Predictions with an E-value ≤ 10-20 are considered reliable (green), even in those cases where the length of the amino acid sequence may be slightly off (yellow).

File Name: Supplementary Data 3
Description: Functional orthologs involved in biosynthetic pathways predicted to be encoded in the studied genomes by HMMs of KO. Predictions with an E-value ≤ 10-20 are considered reliable (green), even in those cases where the length of the amino acid sequence may be slightly off (yellow).

File Name: Supplementary Data 4
Description: Functional orthologs involved in various functions predicted to be encoded in the studied genomes by HMMs of KO. Predictions with an E-value ≤ 10-20 are considered reliable (green), even in those cases where the length of the amino acid sequence may be slightly off (yellow).

File Name: Supplementary Data 5
Description: Phylogenetic tree of groups 1, 2 and 3 NiFe hydrogenases with full bootstrap values as pdf.

File Name: Supplementary Data 6
Description: Groups 1, 2 and 3 NiFe hydrogenases Bayesian phylogeny in newick format.

File Name: Supplementary Data 7
Description: Groups 1, 2 and 3 NiFe hydrogenases alignment.

File Name: Supplementary Data 8
Description: NiFe hydrogenases look up file.

File Name: Supplementary Data 9
Description: FeFe hydrogenases phylogenetic tree with full bootstrap values as pdf.

File Name: Supplementary Data 10
Description: FeFe hydrogenases Bayesian phylogeny in newick format.

File Name: Supplementary Data 11
Description: FeFe hydrogenases alignment.

File Name: Supplementary Data 12
Description: FeFe hydrogenases look up file.

File Name: Supplementary Data 13
Description: Phylogenetic tree of group 4 NiFe hydrogenases and hydrogenaserelated complexes with full bootstrap values as pdf.

File Name: Supplementary Data 14
Description: Group 4 NiFe hydrogenases and hydrogenase-related complexes Bayesian phylogeny in newick format.

File Name: Supplementary Data 15
Description: Group 4 NiFe hydrogenases and hydrogenase-related complexes alignment.

File Name: Supplementary Data 16
Description: V-type ATPase operon gene arrangements in the studied genomes in comparison to Lolkema et al. 46.

File Name: Supplementary Data 17
Description: DMSO reductase superfamily tree in newick format.

File Name: Supplementary Data 18
Description: NifHDK phylogenetic tree with full bootstrap values as pdf.

File Name: Supplementary Data 19
Description: NifHDK phylogenetic tree in newick format.

File Name: Supplementary Data 20
Description: NifHDK alignment.

File Name: Supplementary Data 21
Description: NifHDK look up file.

File Name: Supplementary Data 22
Description: Summary of key genes and protein complexes involved in hydrogen and energy metabolism in Margulisbacteria, Saganbacteria, and Melainabacteria in this study.

File Name: Supplementary Data 23
Description: Cyanobacteria genera included in the gene content comparison with a predicted contamination < 5 % (determined with CheckM 47).

File Name: Supplementary Data 24
Description: Cluster-representatives with the greatest number of different conserved marker genes used to build the species tree of the Terrabacteria.

File Name: Supplementary Data 25
Description: Heme-copper oxygen reductases phylogenetic tree in newick format
