# Supplementary figures and images for: Hydrogen-based metabolism as an ancestral trait in lineages sibling to the Cyanobacteria
Source: Nat Commun. 2019 Jan 28;10:463. doi: 10.1038/s41467-018-08246-y (PMC6349859; doi:10.1038/s41467-018-08246-y)

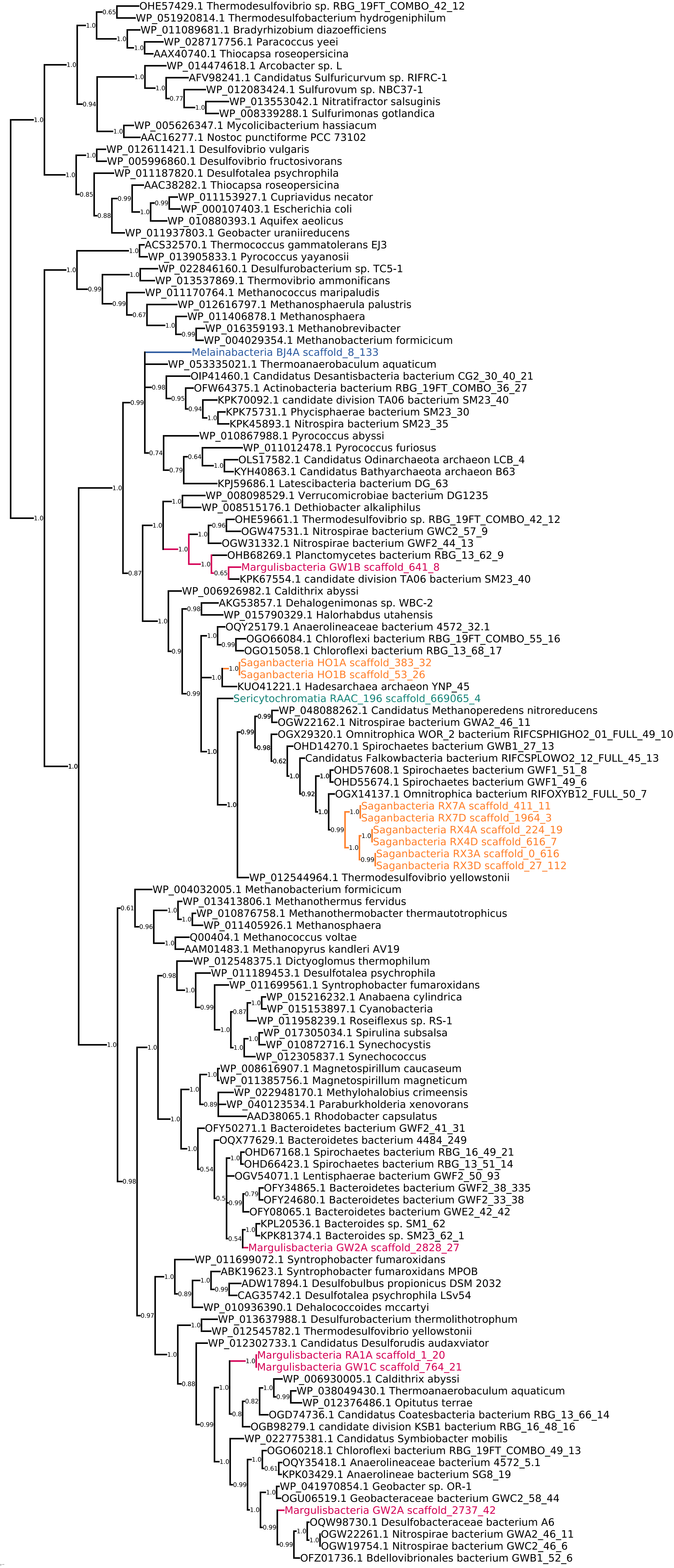

Supplement: Supplementary file 8 — Supplementary Data 5 [file 41467_2018_8246_MOESM8_ESM.pdf]

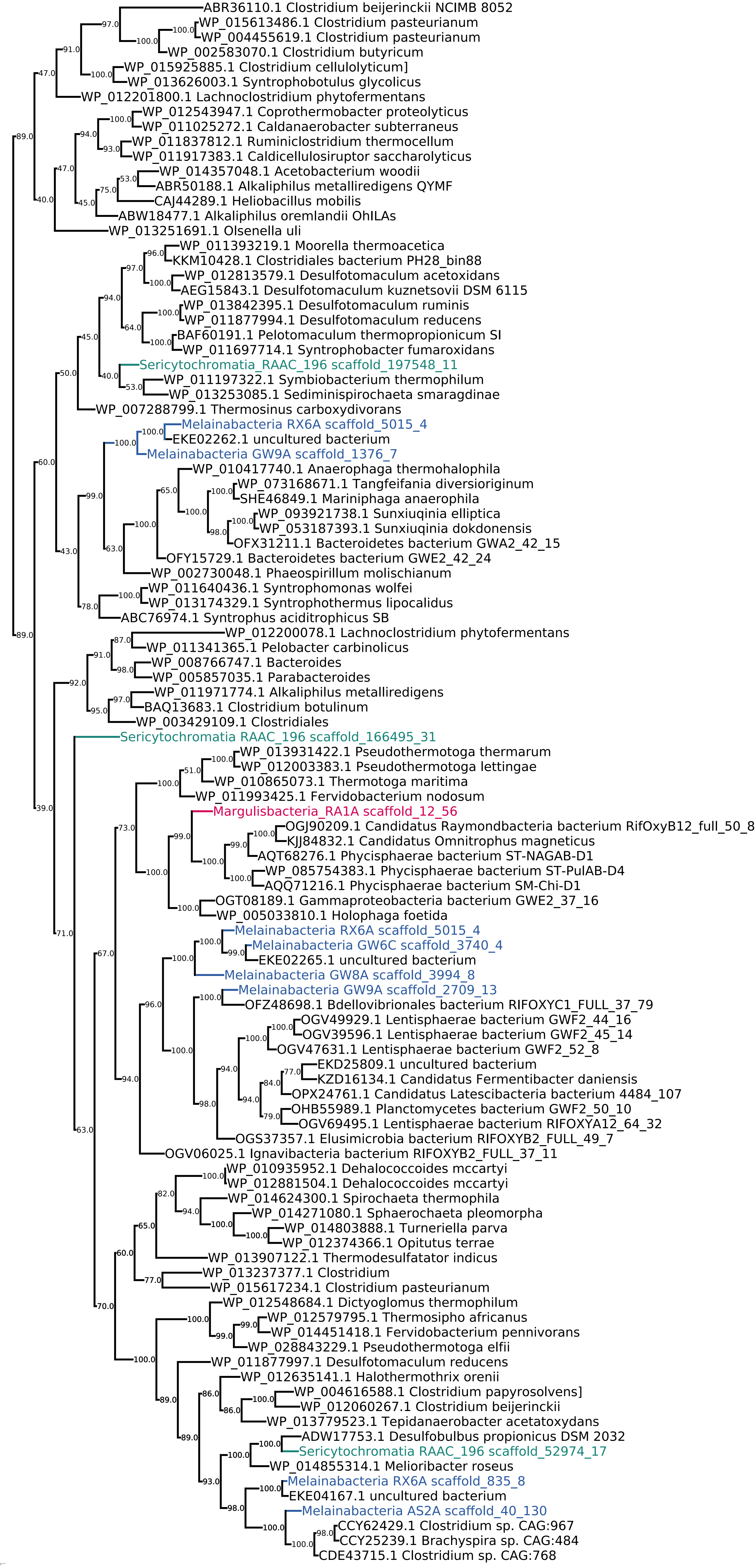

Supplement: Supplementary file 12 — Supplementary Data 9 [file 41467_2018_8246_MOESM12_ESM.pdf]

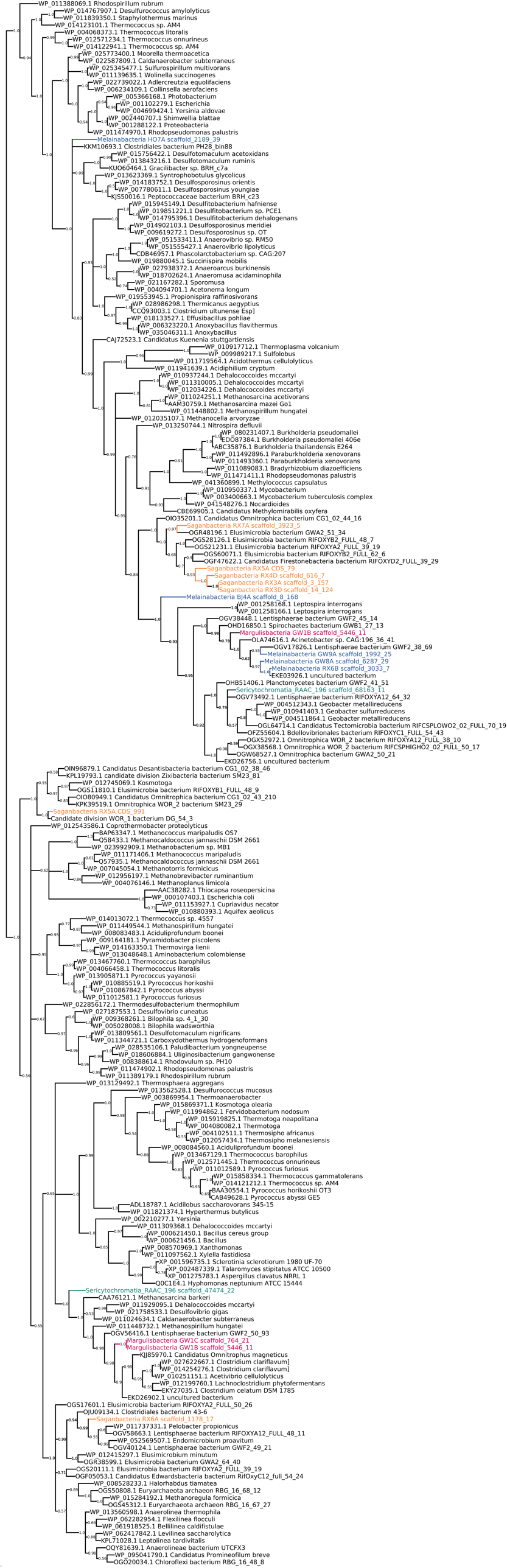

Supplement: Supplementary file 16 — Supplementary Data 13 [file 41467_2018_8246_MOESM16_ESM.pdf]

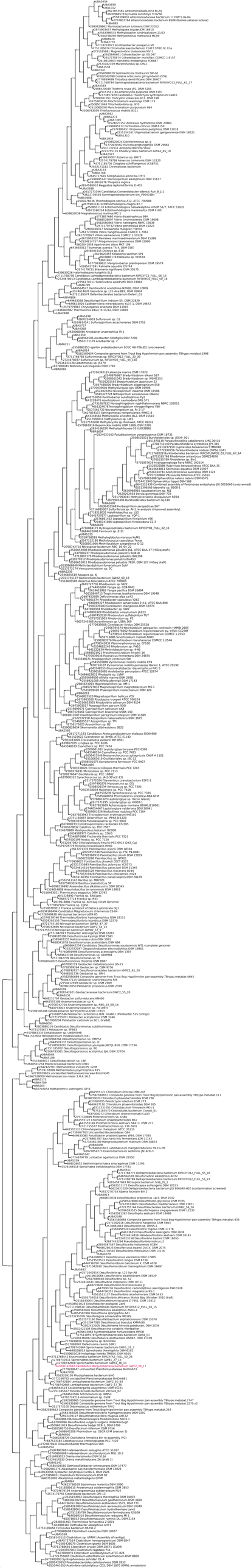

Supplement: Supplementary file 21 — Supplementary Data 18 [file 41467_2018_8246_MOESM21_ESM.pdf]
