# Supplementary material for: Hydrogen-based metabolism as an ancestral trait in lineages sibling to the Cyanobacteria
Source: Nat Commun. 2019 Jan 28;10:463. doi: 10.1038/s41467-018-08246-y (PMC6349859; doi:10.1038/s41467-018-08246-y)
Supplement: Supplementary file 29 — Reporting Summary [file 41467_2018_8246_MOESM29_ESM.pdf]

nature research

Corresponding author(s): Jillian F. Banfield

Reporting Summary

Nature Research wishes to improve the reproducibility of the work that we publish. This form provides structure for consistency and transparency in reporting. For further information on Nature Research policies, see Authors & Referees and the Editorial Policy Checklist.

Statistical parameters

When statistical analyses are reported, confirm that the following items are present in the relevant location (e.g. figure legend, table legend, main text, or Methods section).

n/a	Confirmed	
☒	☐	The exact sample size (*n*) for each experimental group/condition, given as a discrete number and unit of measurement
☒	☐	An indication of whether measurements were taken from distinct samples or whether the same sample was measured repeatedly
☒	☐	The statistical test(s) used AND whether they are one- or two-sided<br>*Only common tests should be described solely by name; describe more complex techniques in the Methods section.*
☒	☐	A description of all covariates tested
☒	☐	A description of any assumptions or corrections, such as tests of normality and adjustment for multiple comparisons
☒	☐	A full description of the statistics including central tendency (e.g. means) or other basic estimates (e.g. regression coefficient) AND variation (e.g. standard deviation) or associated estimates of uncertainty (e.g. confidence intervals)
☒	☐	For null hypothesis testing, the test statistic (e.g. *F*, *t*, *r*) with confidence intervals, effect sizes, degrees of freedom and *P* value noted<br>*Give P values as exact values whenever suitable.*
☐	☒	For Bayesian analysis, information on the choice of priors and Markov chain Monte Carlo settings
☒	☐	For hierarchical and complex designs, identification of the appropriate level for tests and full reporting of outcomes
☒	☐	Estimates of effect sizes (e.g. Cohen's *d*, Pearson's *r*), indicating how they were calculated
☒	☐	Clearly defined error bars<br>*State explicitly what error bars represent (e.g. SD, SE, CI)*

Our web collection on statistics for biologists may be useful.

Software and code

Policy information about availability of computer code

Data collection	No software was used for the specific purpose of data collection.
Data analysis	Sickle v1.33 (https://github.com/najoshi/sickle)<br>IDBA_UD v1.1.0<br>ESOM<br>prepare_esom_files.pl (https://github.com/CK7/esom/blob/master/prepare_esom_files.pl)<br>DASTool v1.0<br>CONCOCT v0.4.1<br>ABAWACA v1.00 (https://github.com/CK7/abawaca)<br>MaxBin v2.2<br>ggKbase (https://ggkbase.berkeley.edu/)<br>fix_assembly_errors (https://github.com/christophertbrown/fix_assembly_errors/releases/tag/2.00)<br>Bowtie2<br>Geneious v9.0.5<br>SPAdes v3.9.0<br>Prodigal v2.6.3<br>BLAST<br>hmmsearch<br>Interpro (https://www.ebi.ac.uk/interpro/)

CD search
OrthoFinder v2.1.3
FastANI v1.0
CheckM v1.0.11
MAFFT v7.294b
BMGE v1.1
IQ-tree (multicore v1.5.5)
PhyloBayesMPI v1.7
hmmer v3.1b2
trimal v1.4
Muscle v3.8.31
RAxML-HPC BlackBox v8.2.10
CIPRES Science Gateway (http://www.phylo.org/index.php/)
Microsoft Excel v11

For manuscripts utilizing custom algorithms or software that are central to the research but not yet described in published literature, software must be made available to editors/reviewers upon request. We strongly encourage code deposition in a community repository (e.g. GitHub). See the Nature Research guidelines for submitting code & software for further information.

Data

Policy information about availability of data

All manuscripts must include a data availability statement. This statement should provide the following information, where applicable:
- Accession codes, unique identifiers, or web links for publicly available datasets
- A list of figures that have associated raw data
- A description of any restrictions on data availability

New and published genomes included in this study and corresponding gene annotations can be accessed at https://ggkbase.berkeley.edu/Margulis_Sagan_Melaina/ organisms (ggKbase is a 'live' site, genomes may be updated after publication). DNA sequences (new genomes and raw sequence reads) have been deposited in the NCBI Bioproject Database (accession codes: PRJNA167727 [https://www.ncbi.nlm.nih.gov/bioproject/PRJNA167727], PRJNA451230 [https://www.ncbi.nlm.nih.gov/bioproject/?term=PRJNA451230], PRJNA471730 [https://www.ncbi.nlm.nih.gov/bioproject/?term=PRJNA471730], PRJNA471718 [https://www.ncbi.nlm.nih.gov/bioproject/?term=PRJNA471718]). Further details are provided in Supplementary Table 1, including NCBI Genbank accession numbers for individual genomes. The source data underlying Figs. 1, 4, 5, 6, and 7 and Supplementary Fig. 4 are provided as Source Data files. A reporting summary for this article is available as a Supplementary Information file.

Field-specific reporting

Please select the best fit for your research. If you are not sure, read the appropriate sections before making your selection.

☒ Life sciences ☐ Behavioural & social sciences ☐ Ecological, evolutionary & environmental sciences

For a reference copy of the document with all sections, see nature.com/authors/policies/ReportingSummary-flat.pdf

Life sciences study design

All studies must disclose on these points even when the disclosure is negative.

Sample size	We gathered publicly available metagenome-assembled genomes (MAGs) members of the Margulisbacteria (4), Saganbacteria (26), and Melainabacteria (8) whose genomes had not been described in other studies. Additionally, 1 newly generated sediment-associated Margulisbacteria, 7 single amplified ocean-associated Margulisbacteria genomes, and 5 Melainabacteria MAGs were compiled for the present study.
Data exclusions	1 Melainabacteria MAG and 4 ocean-associated Margulisbacteria SAGs of low quality as defined by the standards for minimum information about a metagenome-assembled genome (MIMAG) and a single amplified genome (MISAG) were excluded from the metabolic potential analyses.
Replication	In most cases reproducibility was obtained in the form of multiple genomes with average nucleotide identity > 97%.
Randomization	Control of covariates was not relevant for this study. Genomes were analyzed independently from each other and only grouped by taxonomic affiliation.
Blinding	Blinding was not relevant for this study.

Reporting for specific materials, systems and methods

Materials & experimental systems

n/a	Involved in the study
☒	☐ Unique biological materials
☒	☐ Antibodies
☒	☐ Eukaryotic cell lines
☒	☐ Palaeontology
☒	☐ Animals and other organisms
☐	☒ Human research participants

Methods

n/a	Involved in the study
☒	☐ ChIP-seq
☒	☐ Flow cytometry
☒	☐ MRI-based neuroimaging

Human research participants

Policy information about studies involving human research participants

Population characteristics	Not relevant for this study, because the analyses conducted were exclusively related to the functional potential of bacterial genomes regardless of their origin.
Recruitment	Not relevant for this study, because the analyses conducted were exclusively related to the functional potential of bacterial genomes regardless of their origin.
